# 100. Assessment of Emergency Department Prescribing Practices for Outpatient Treatment of Urinary Tract Infection, Community-Acquired Pneumonia, and Skin and Soft Tissue Infections

**DOI:** 10.1093/ofid/ofab466.302

**Published:** 2021-12-04

**Authors:** Matthew Thaller, Casey J Dempsey, Alexander Levine, Kelly Shepard

**Affiliations:** 1 The Hospital of Central Connecticut, South Windsor, Connecticut; 2 Hartford HealthCare, Bolton, Connecticut

## Abstract

**Background:**

Studies have found a need for improved antimicrobial stewardship in the outpatient setting. The literature is limited by the populations and disease states studied as many focus on viral infections. This study focuses on the adult emergency departments (EDs) in a large healthcare system and quantifies the proportion of antibiotic prescriptions deemed inappropriate for common outpatient infections.

**Methods:**

A retrospective study was conducted in patients with selected common infections treated as an outpatient from the ED. Patients were reviewed for eligibility based on the inclusion and exclusion criteria in Table 1. Appropriateness was analyzed based on: need for antimicrobial therapy; agent choice, dose, duration, and directions in concordance with national guidelines and local resistance patterns; and no clinically relevant drug interactions, unnecessary dual coverage, or a better or safer alternative available. The entire prescription was marked inappropriate if any factor was deemed inappropriate.

Table 1. Inclusion and Exclusion Criteria

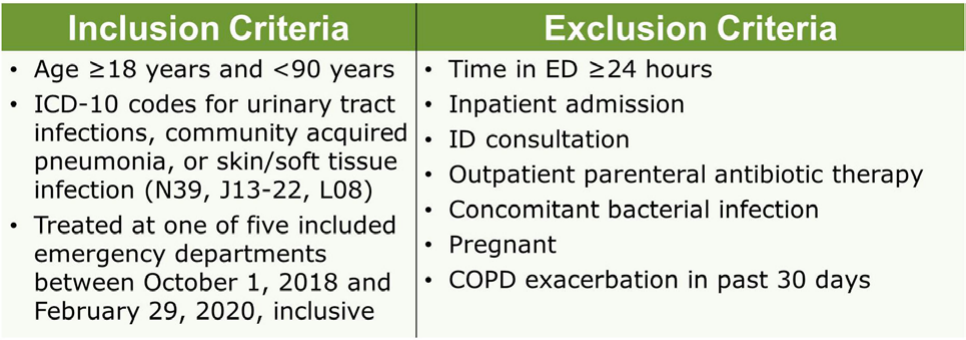

Based on the Epic report generated, a random sample of patients were selected for manual review. Only patients who met the following criteria were eligible for inclusion in the final analysis.

**Results:**

Of the 318 patients reviewed, 274 were included. Treatment was deemed inappropriate 64% (174/274) of the time, significantly above the estimated 30% (p < 0.001). The agent selection, duration, and dose were the most the frequent factors deeming a prescription inappropriate. The most inappropriately used agents were fluoroquinolones and azithromycin. A positive culture required modification of therapy 31% (22/70) of the time and more so when the drug was guideline recommended. For example, when empiric antibiotic selection was per urinary tract infection guidelines, 31% (14/53) required modification compared to 19% (3/16) when the agent was not. This was most apparent when cephalexin was used.

**Conclusion:**

The use of antibiotics at the studied EDs was not in concordance with guidelines in the study period. However, the cultures were sensitive less often to agents deemed appropriate per guidelines for empiric therapy. It is possible that the ideal treatments of bacterial infections in this community are not representative of national resistance patterns. Using ED-specific antibiograms to create order panels for common infections, as well as prospective pharmacist review at ED discharge, could increase appropriate utilization of preferred agents.

**Disclosures:**

**All Authors**: No reported disclosures

